# Clinical course of acute zonal occult outer retinopathy complicated by choroidal neovascularization

**DOI:** 10.1186/s40942-018-0134-y

**Published:** 2018-08-29

**Authors:** Ugo Introini, Giuseppe Casalino, Elona Dhrami-Gavazi, Sri Krishna Mukkamala, Sarah Mrejen, Hermann Schubert, Salomon Y. Cohen, Claudio Azzolini, Francesco Bandello, Stanley Chang, Lawrence A. Yannuzzi

**Affiliations:** 1 Department of Ophthalmology, San Raffaele Scientific Institute, Vita-Salute University, Milan, Italy; 2 Moorfields Eye Hospital, NHS Foundation Trust, London, UK; 3 Vitreous Retina Macula Consultants of New York, New York, USA; 4 Department of Ophthalmology, Edward S. Harkness Eye Institute, Columbia University College of Physicians and Surgeons, New York, USA; 5 Ophthalmic Center for Imaging and Laser, Paris, France; 6 Department of Surgical Sciences, Ophthalmology Section, University of Insubria Ospedale di Circolo, Varèse, Italy

**Keywords:** Acute zonal occult outer retinopathy, Choroidal neovascularization, Anti-vascular endothelial growth factor treatment, Multimodal retinal imaging

## Abstract

**Purpose:**

To report the clinical course and multimodal imaging features of acute zonal occult outer retinopathy (AZOOR) complicated by choroidal neovascularization (CNV) treated with anti-vascular endothelial growth factor (VEGF) treatment or photodynamic therapy (PDT).

**Methods:**

Observational case series. Retrospective analysis of patients presenting to different institutions with evidence of AZOOR and neovascular lesions. Diagnosis of AZOOR was made on the basis of clinical presentation and multimodal imaging. All patients underwent a comprehensive ophthalmic evaluation and multimodal retinal imaging, including color fundus photos, fundus autofluorescence, fundus fluorescein angiography and spectral-domain optical coherence tomography.

**Results:**

Four patients (three males, mean age 53.5 years) were included in the study. Mean follow-up was 5.1 years. Presentation of AZOOR was unilateral in two patients and bilateral in the remainder two patients. One of the patients presenting with unilateral AZOOR developed zonal lesions in the fellow eye during follow-up. All patients presented with unilateral type 2 (subretinal) CNV. Three patients underwent intravitreal anti-VEGF injections and one patient underwent a single PDT. Multimodal retinal imaging showed zonal or multizonal progression during treatment. After treatment, visual acuity and CNV stabilization was observed in all patients.

**Conclusions:**

The presence of CNV expands the clinical spectrum of AZOOR. CNV complicating AZOOR may be effectively treated with intravitreal injections of anti-VEGF, despite progression of the zonal lesions. Further studies are required to define the role of treatment in the progression of the zonal lesions.

## Background

As originally defined in 1992 by Gass in his Donder’s Lecture [1], acute zonal occult outer retinopathy (AZOOR) is a rare, condition of unknown etiology, characterized by an acute loss of one or more zones of outer retinal function. Since the original report by Gass [1], the term AZOOR became a general diagnostic term for chorioretinal diseases with visual loss of uncertain origin. Indeed, the definition and classification of AZOOR resulted in a heterogenic spectrum of disorders, the so-called “AZOOR complex”.

A common feature of the disease is the acute zonal lesion, or lesions, delineated by a grayish ring on funduscopy or a demarcation line of hyperautofluorescence seen on fundus autofluorescence imaging, which has been referred to as acute annular outer retinopathy (AAOR) [2]. A more strict definition of AZOOR based on multimodal retinal imaging findings, including a demarcating line of progression between the involved and uninvolved retina and a trizonal pattern of chorioretinal degeneration, has been recently proposed [3, 4].

The development of subretinal choroidal neovascularization (CNV) in AZOOR is an extremely rare event [4–6]. Levison et al. [6] recently reported a case of CNV in AZOOR but long-term follow-up was not included. The purpose of this study is to report in details the clinical course and the multimodal retinal imaging of patients presenting with CNV and AZOOR.

## Methods

This is an observational case series of patients included from the practices of different retina specialists. Clinical records and multimodal retinal imaging of patients presenting with AZOOR and neovascular lesions from three tertiary referring centers located in Italy (San Raffaele Scientific Institute, Vita-Salute University, Milan), France (Ophthalmic Center for Imaging and Laser, Paris, France) and United States of America (Vitreous Retina Macula Consultants of New York, New York) were collected and analysed. All patients underwent a comprehensive ophthalmic evaluation, including best-corrected visual acuity (BCVA), and multimodal retinal imaging, including color fundus photography, fundus autofluorescence (FAF), fundus fluorescein angiography (FA) and spectral-domain optical coherence tomography (SD-OCT). Indocyanine green angiography (ICGA) was available for 3 patients (cases 2, 3 and 4).

Wide-field imaging using a system imaging 200° of the retina (200Tx; Optos plc) was available for one patient (case 3). For the other patients FAF, FA and ICGA were obtained using a fundus camera (TRC 501; Topcon Medical Systems) or scanning laser ophthalmoscopy on the Heidelberg system (Heidelberg Engineering, Heidelberg).

Diagnosis of AZOOR was made on the basis of clinical presentation and multimodal retinal imaging features including: (1) a demarcating line of progression at the level of the outer retina; (2) a trizonal pattern of sequential involvement of the outer retina, retinal pigment epithelium (RPE) and choroid; (3) zonal progression [4].

Extensive laboratory testing was performed in all patients to rule out chorioretinal conditions mimicking AZOOR. Humphrey visual field test was performed in two patients (cases 1 and 2) and Goldmann dynamic perimetry in one patient (case 4).

Demographic and relevant clinical findings available at baseline and at all follow-up examination visits were reviewed by the authors and retrospectively analyzed. Early data from one of the included cases (case 4) have been reported by Cohen and Jampol [5].

The study was conducted in agreement with the Declaration of Helsinki for research involving human subjects. Informed consent for publication of their clinical details and/or clinical images was obtained from the patients.

## Results

Four patients (3 males) were included in this study. Two patients attended the Vitreous Retina Macula Consultants of New York (cases 1 and 3); one patient attended the San Raffaele Scientific Institute in Milan (case 2); one patient attended the Ophthalmic Center for Imaging and Laser in Paris (case 4).

Demographic characteristics, main presenting symptoms and medical history of patients are shown in Table 1. The main presenting clinical characteristics of patients are shown in Table 2. The characteristics of a published case of AZOOR complicated by CNV [6] is reported in Tables 1 and 2.

**Table 1:** Demographic characteristics, main presenting symptoms and medical history of acute zonal occult outer retinopathy complicated by chorodal neovascularization in our series and in the Levison et al.^a^ report

Patient	Gender	Age	Presenting symptoms	Medical history
1	M	65	Central scotoma	Systemic hypertension; aortic valve replacement
2	M	69	Blurred vision, scotoma, photopsia	Systemic hypertension
3	F	33	Central scotoma and distortion	Hashimoto’s thyroiditis; ashtma
4	M	47	Blurred vision and photopsia	Unremarkable
Levison et al.^a^	F	74	Blurred vision, scotoma, photopsia	Not reported

*M* male, *F* female

^a^See Ref. [6]

**Table 2:** Clinical characteristics of acute zonal occult outer retinopathy complicated by chorodal neovascularization (CNV) in our series and in the Levison et al.^a^ report

Patient	Presentation	Eye with CNV	CNV treatment	N tretments	FU (years)	Final BCVA
1	Unilateral	LE	Beva	3	4	RE 20/20 LE 20/60
2	Bilateral	LE	Beva	3	2	RE 20/25 LE 20/50
3	Bilateral	RE	Rani + Afli	12 + 5	3	RE 20/40 LE 20/20
4	Unilateral	LE	PDT	1	11	RE 20/40^b^ LE 20/20
Levison et al.^a^	Bilateral	RE	Beva	3	0.4	RE 20/20 LE HM

*RE* right eye, *LE* left eye, *Beva* bevacizumab, *Rani* Ranibizumab, *Afli* aflibercept, *PDT* photodynamic therapy, *FU* follow-up, *BCVA* best-corrected visual acuity

^a^See Ref. [6]

^b^With eccentric vision

In our series, mean age of the patients at the time of presentation was 53.5 year-old, ranging from 33 to 69 year-old. Central scotoma and photopsia were the most common presenting symptoms. AZOOR was unilateral in two patients (cases 1 and 4) and bilateral in the remainder two patients (cases 1 and 4). Mean follow-up was 5.1 years. One of the patients presenting with unilateral AZOOR developed zonal lesions in the fellow eye 5 years after presentation (case 4).

All patients presented with unilateral type 2 (subretinal) CNV which was treated with intravitreal anti-VEGF injections in 3 patients (bevacizumab in two patients; ranibizumab and aflibercept in one patient) and Verteporfin photodynamic therapy (PDT) in one patient (case 4). After treatment, visual acuity and CNV stabilization was observed in all patients. Multimodal retinal imaging showed progression of the zonal lesions during treatment.

### Case 1

**Fig. 1:**
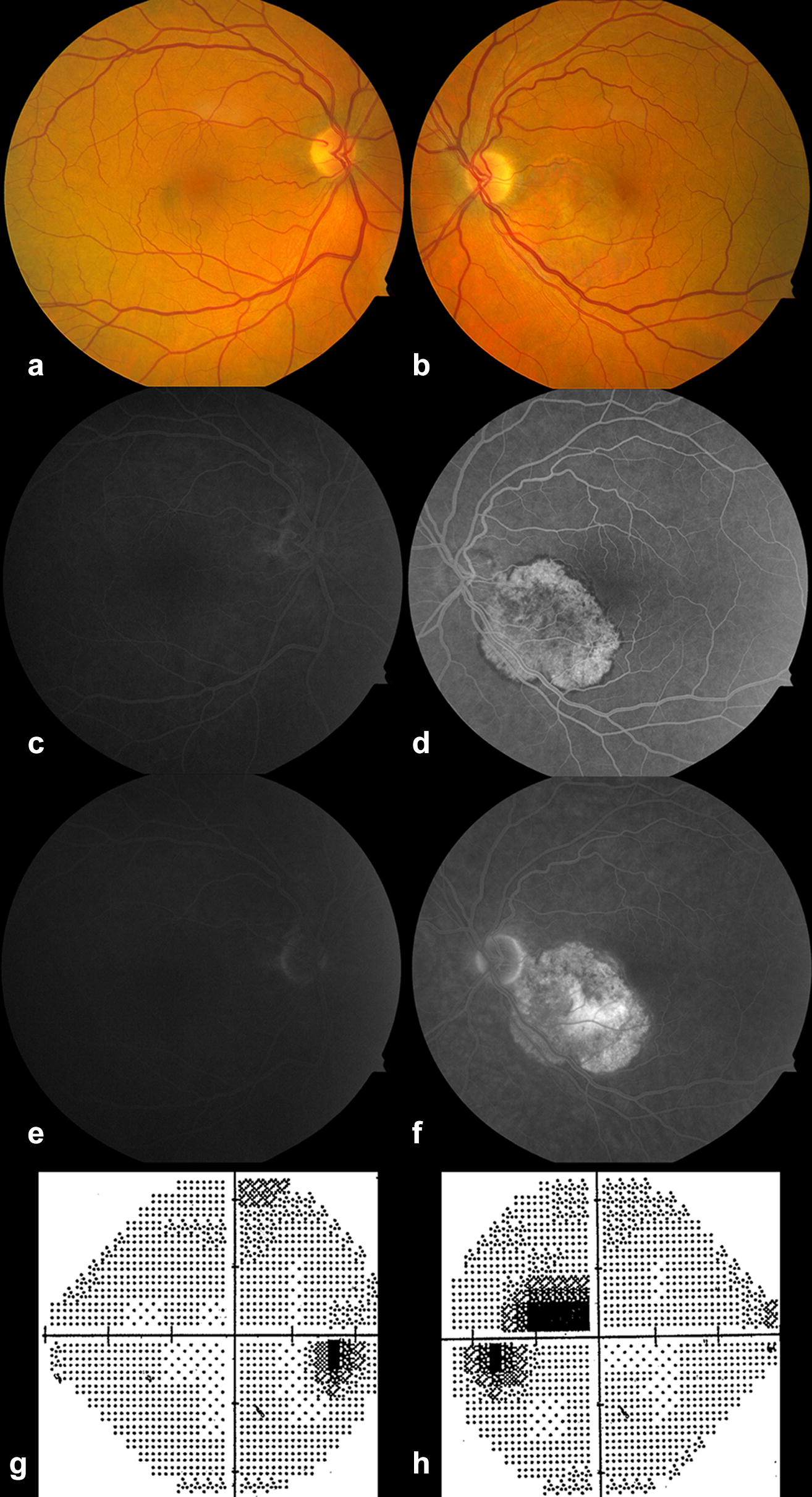
Case 1. **a**, **b** Color fundus photos at presentation. **a** The right fundus was found to be normal; **b** the left fundus revealed a well-delineated area of retinal pigment epithelium atrophy in the inferonasal macula with foveal sparing. **c**, **e** Early and late phase fluorescein angiography images of the right eye were normal. **d**, **f** In the left eye, there was early diffuse hyperfluorescence with a hypofluorescent border. In late phase, there was late staining of a patch in the center of the lesion. **g**, **h** Humphrey visual field 24-2 revealed a full visual field in the right eye and a superior paracentral scotoma corresponding to the lesion in the left eye

**Fig. 2:**
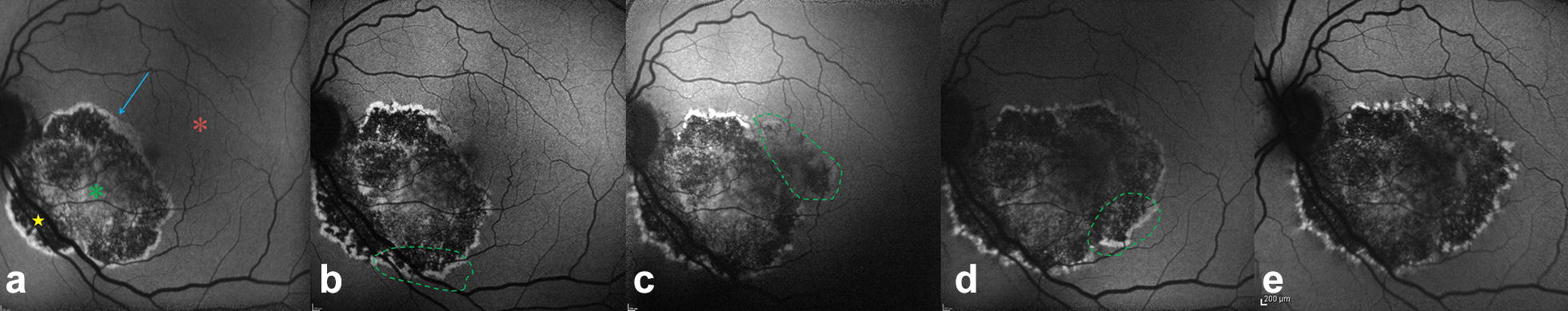
Case 1. Fundus autofluorescence (FAF) at presentation (**a**), day 10 (**b**), week 3 (**c**), month 2 (**d**), and month 5 (**e**). At presentation (**a**), there was a trizonal FAF pattern. There was an area of diffuse hypoFAF (yellow star) surrounded by a large area of speckled FAF (green asterisk). These areas were circumscribed by a continuous hyper-FAF demarcation line (blue arrow) outside of which the retina appeared normal (red asterisk). In **b**–**d**, there was FAF evidence of lesion expansion in areas encircled by the green dashed line. Of note, in 3C, there is no demarcation line around the area of new expansion, possibly indicating a very recent change. Between month 2 and month 5, there was minimal evidence of lesion change

A 65-year-old white male presented 10 days after the onset of a central scotoma in the left eye (LE). BCVA at presentation was 20/20 in the right eye (RE) and 20/32 in the LE. Fundus examination of the LE revealed an area of RPE atrophy in the inferonasal macula with foveal sparing (Fig. 1). FAF in the left eye revealed a trizonal pattern and a demarcating hyper-FAF line between the involved and uninvolved retina (Fig. 2); these findings were consistent with AZOOR [4].

**Fig. 3:**
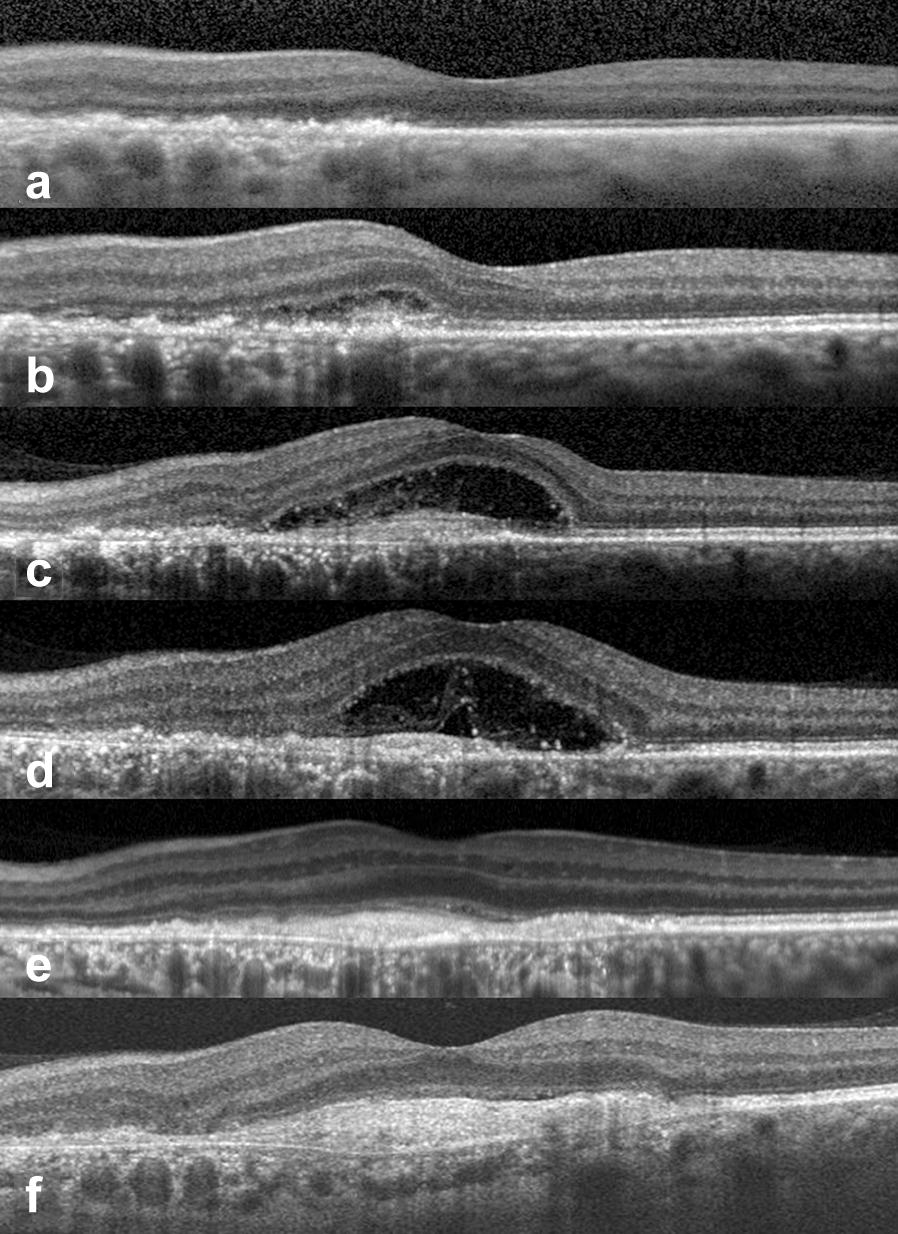
Case 1. Spectral-domain optical coherence tomography images of the left eye at presentation (**a**), day 10 (**b**), week 3 (**c**), week 6 (**d**), week 10 (**e**), and month 5 (**f**). At presentation (**a**) there was evidence of normal foveal contour with outer retinal changes disrupting the continuity of the ellipsoid zone band. At day 10 (**b**), there was mild subretinal fluid nasal to the fovea. At week 3 (**c**), there was a significant increase in the quantity of subretinal fluid. There was evidence of hyperreflective material within the subretinal fluid that may represent fibrin. Below the subretinal fluid, there appeared to be layered hyperreflective bands which could represent the layering of the fibrin. By week 6 (**d**), there was more fluid, and a consolidation of the hyperreflective material. By week 10 and month 5, there is resolution of the subretinal fluid with persistent well-defined hyperreflective material consistent with retinal scar

Ten days later, he returned complaining of distortion. Fundus examination revealed zonal lesion expansion, which was confirmed by FAF. The OCT revealed mild subretinal fluid (SRF) which explained his metamorphopsia. The patient returned a week later. At this time, visual acuity had reduced to 20/60, and two perilesional haemorrhages were observed along with a hypo-FAF zonal lesion expansion which included the fovea (Fig. 2). The OCT showed a significant increase of subretinal fluid. These findings were consistent with a type 2 (subretinal) CNV which was confirmed by fluorescein angiography. Consequently he received 3 monthly intravitreal bevacizumab injections. One month after the first injection, FAF revealed zonal progression (Fig. 2) and OCT revealed a worsening of SRF (Fig. 3). One month after the third injection, BCVA in the LE was 20/70 and the patient reported stabilization of his central scotoma. At month 5, BCVA was 20/60; OCT scan showed resolution of SRF, while FAF revealed minimal advancement of zonal lesion size. During follow-up the zonal lesions stabilized; the patient received 11 intravitreal bevacizumab injections on a treat and extend regimen for 4 years and maintained a BCVA of 20/60 in the LE.

### Case 2

**Fig. 4:**
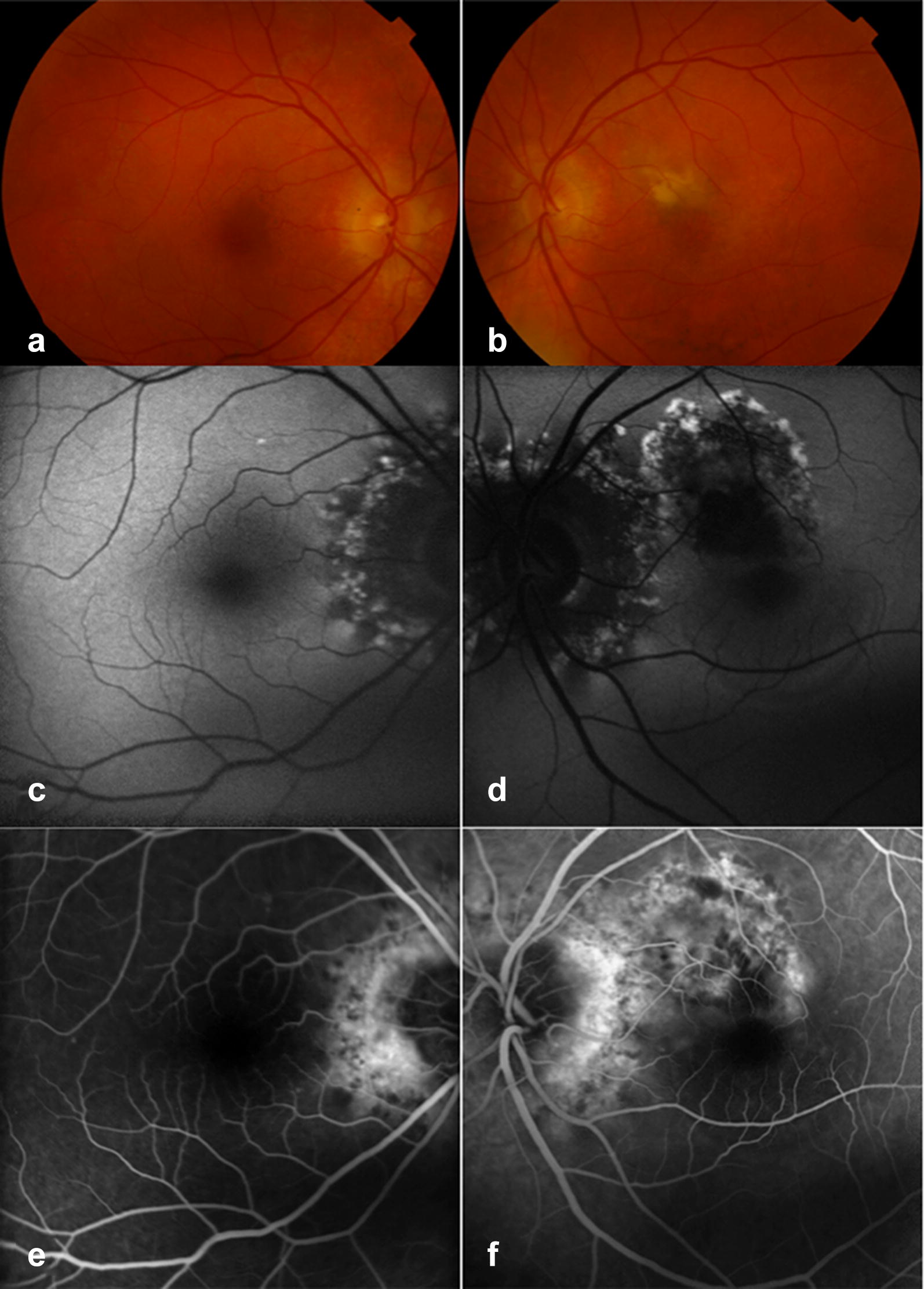
Case 2. Multimodal retinal imaging at presentation. **a**, **b** Fundus examination revealed bilateral peripapillary atrophy with drusen-like material and retinal swelling at the macula of the left eye (LE). **c**, **d** Fundus autofluorescence (FAF) showed bilateral zonal peripapillary hypoFAF areas surrounded by a granular patchy hyper-FAF border. In the LE (**d**), a zonal defect with similar characteristics was detected superior to the fovea. **e**, **f** Fluorescein angiography (FA) showed bilateral peripapillary hyperfluorescence. **f** FA of the LE showed dye leakage from a juxtafoveal type 2 choroidal neovascularizarition

**Fig. 5:**
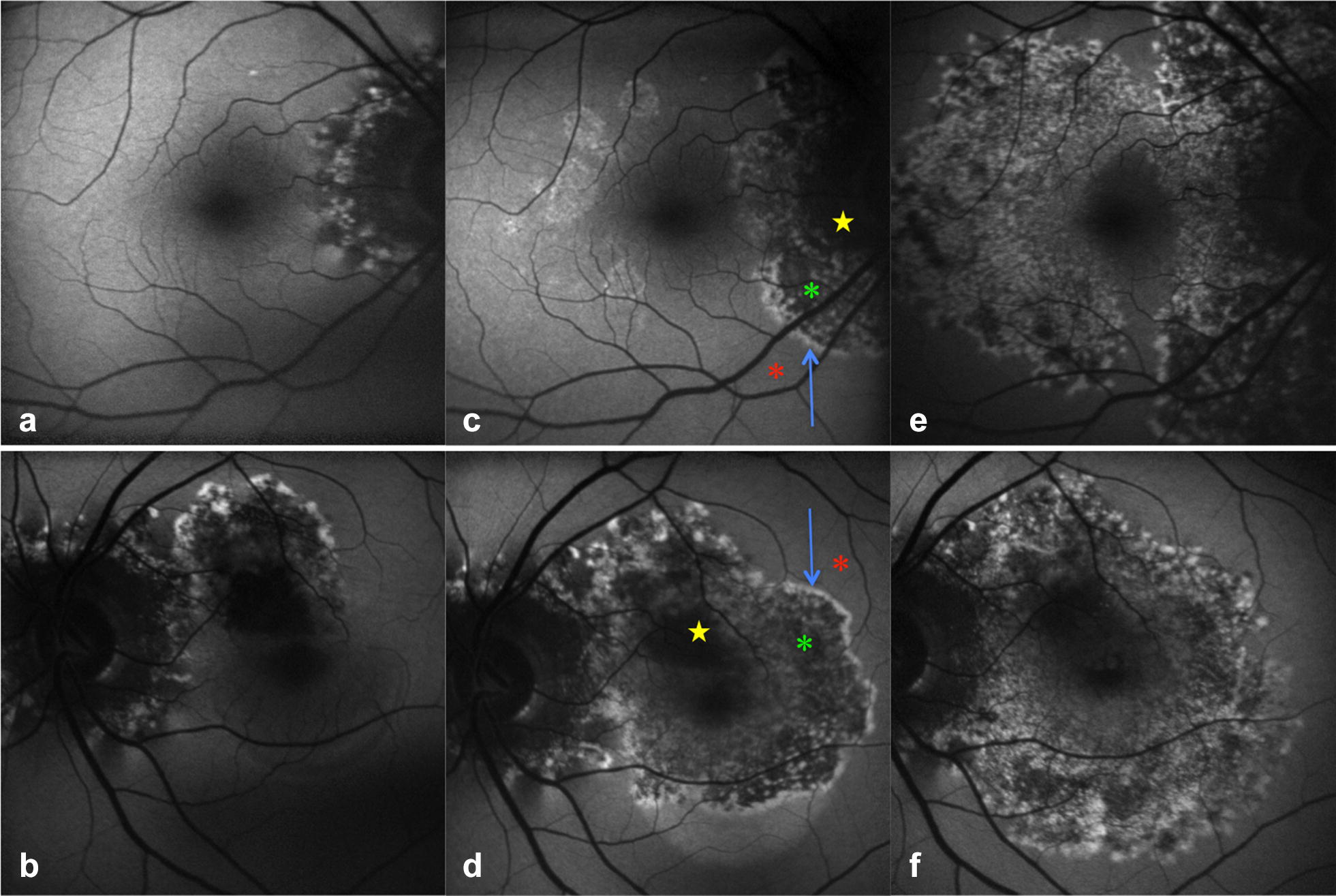
Case 2. Fundus autofluorescence (FAF) at presentation (**a**, **b**), 1 month after 1 anti-VEGF injection (**c**, **d**) and 1 month after 3 anti-VEGF injections (**e**, **f**). **c**, **d** After the first injection, bilateral zonal progression was noted. FAF showed the typical trizonal pattern consisting of an hypo-FAF area (yellow stars) surrounded by a large area of speckled FAF (green asterisks), circumscribed by a continuous annular hyper-FAF demarcation line (blue arrows) at the junction between the involved and uninvolved retina (red asterisks). **c** In the right eye (RE) FAF showed enlargement of the peripapillary zonal lesion and new hyperFAF zonal lesions appeared temporal to the fovea. **d** In the left eye (LE) FAF showed progression of the zonal macular lesion with foveal involvement. **e**, **f** After the third injection FAF showed bilateral progression of the zonal lesions with confluence of the lesions in the RE (**e**) and foveal sparing. In the LE (**f**), the hyper-FAF annular outer border lesion was no longer detectable

A 69-year-old white male was referred for sudden onset scotoma with blurriness and photopsia in the LE. BCVA was 20/20 in the RE, and 20/40 in the LE. Fundus examination revealed bilateral peripapillary atrophy and retinal swelling at the macula of the LE (Fig. 4). OCT scan and FA revealed a juxtafoveal type 2 CNV in the LE. One intravitreal bevacizumab injection was administered in the LE. Four 4 weeks after the injection, BCVA was stable; in the RE the peripapillary lesion extended and new zonal lesions were visible temporal to the fovea (Fig. 5); in the LE the peripapillary and the macular defect extended and merged appearing as a single, large zonal defect involving the fovea. FAF in both eyes revealed a trizonal pattern and a demarcating hyper-FAF line between the involved and uninvolved retina (Fig. 5); these findings were consistent with AZOOR [4].

**Fig. 6:**
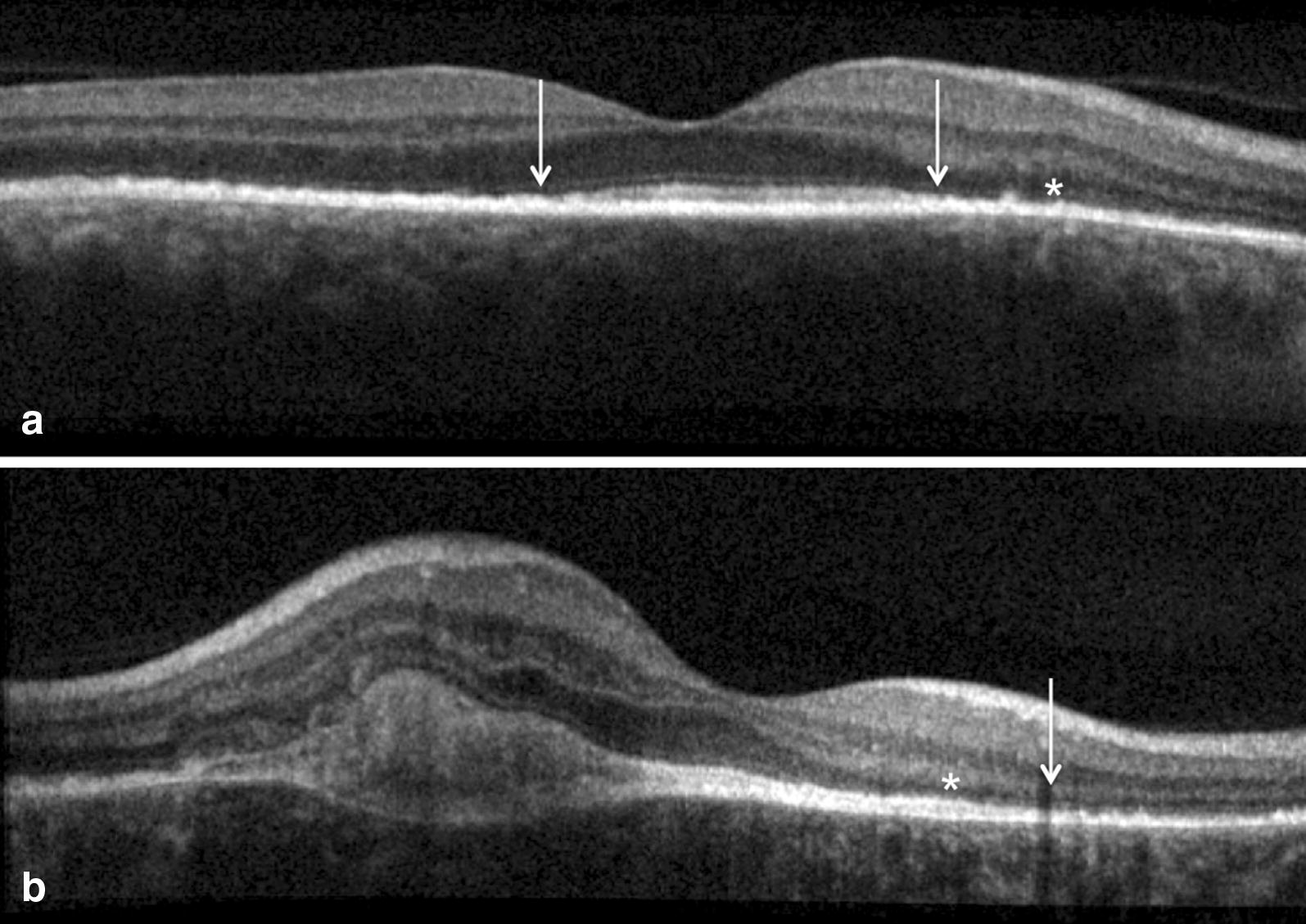
Case 2. Spectral-domain optical coherence tomography (SD-OCT) scan after 3 anti-VEGF injections in the left eye. **a**, **b** SD-OCT scan of right eye (**a**) and left eye (**b**) revealed photoreceptor dysfunction by showing disruption of the ellipsoid zone (white arrows) with thinning of the outer nuclear layer (white asterisks). **b** SD-OCT scan of the left eye showed presence of well-defined hyperreflective material consistent with retinal scar

FA showed persistence of leakage from the CNV and two additional intravitreal bevacizumab injections were administered. One month after the third injection, further progression of the zonal lesions was observed in both eyes (Fig. 5). SD-OCT scan showed complete resolution of the subretinal fluid with persistent well-defined subretinal hyperreflective material (Fig. 6). Since then, the patient has maintained a BCVA of 20/50 in the LE (and 20/25 in the RE) with no evidence of progression of the zonal lesions and CNV stabilization during 2 years of follow-up.

### Case 3

**Fig. 7:**
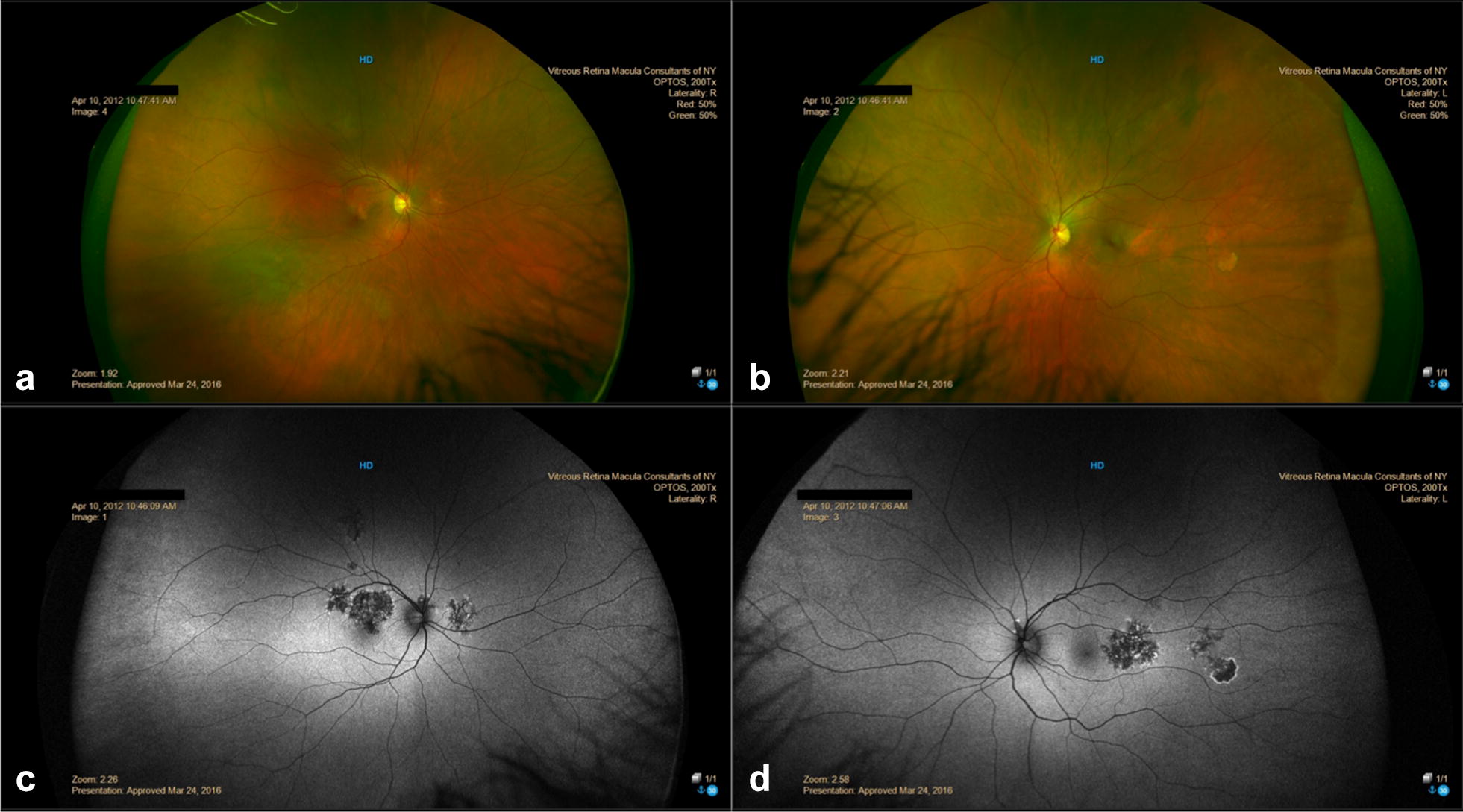
Case 3. Wide field color image and wide field fundus autofluorescence (FAF) at presentation. **a**, **b** Wide field color image showed multiple, well-demarcated zonal areas of outer retinal atrophy that appeared slightly depigmented, with an increased visualization of the major underlying choroidal vessels. **a** In the right eye, there was one main zonal area in the posterior pole and two additional areas in the nasal and the supero-temporal mid-periphery. **b** In the left eye three zonal areas were located at the posterior pole and the temporal mid-periphery. **c**, **d** FAF showed hypo-FAF with granular hyper-FAF signal in correspondence of these depigmented areas. A leading border of hyper-FAF was evident at the junction between the involved and uninvolved retina

**Fig. 8:**
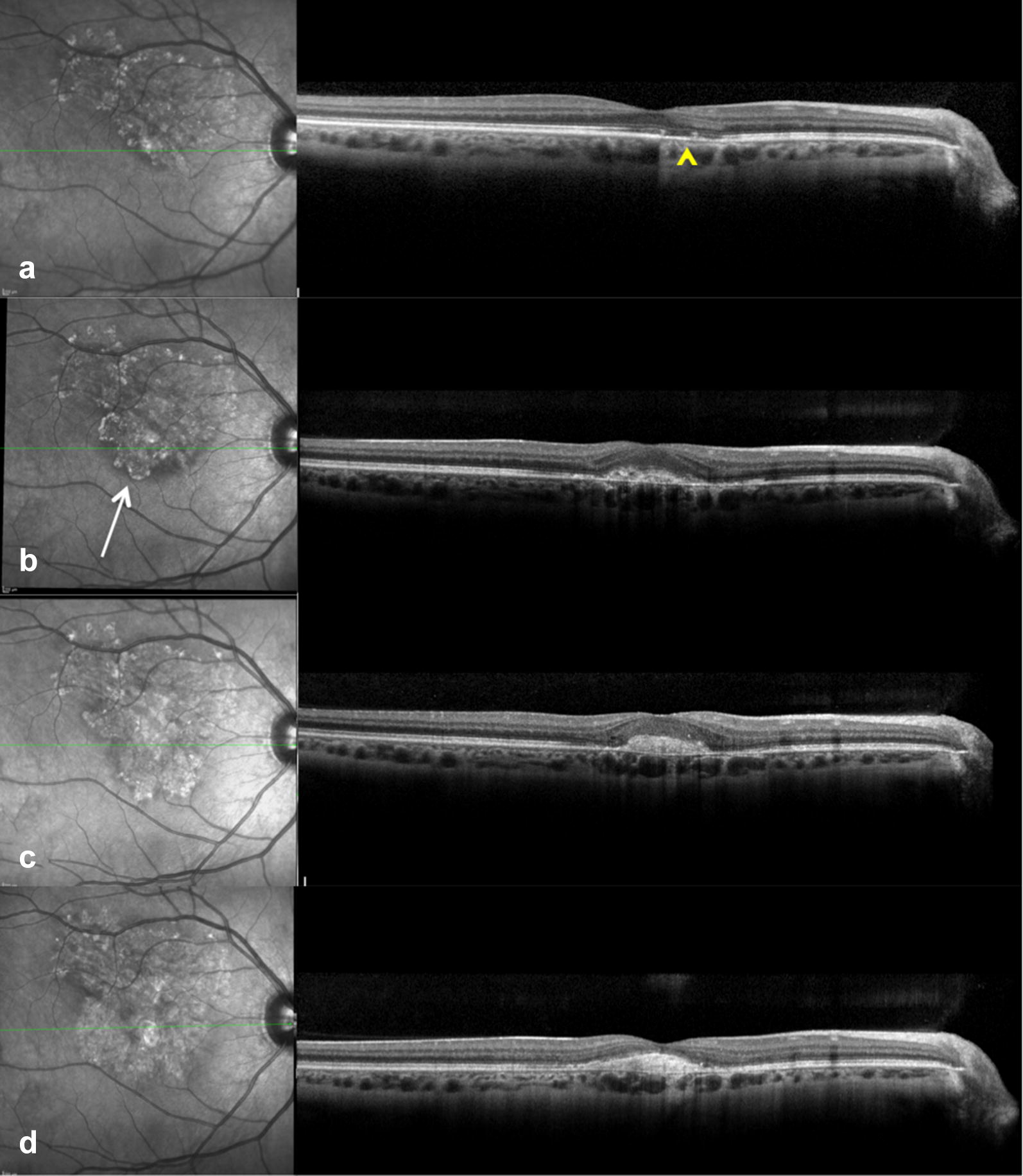
Case 3. Near infrared reflectance (NIR) imaging acquired simultaneously with spectral domain optical coherence tomography (SD-OCT) at presentation and during treatment of the right eye. **a** At presentation SD-OCT scan at the level of the zonal lesion showed focal interruption of the ellipsoid zone (yellow arrowhead). **b** After 2 months NIR imaging showed progression of the zonal lesion (white arrow) and SD-OCT scan showed increased retinal thickness and ill-defined hyperreflective material consistent with a type 2 choroidal neovascularization. **c**, **d** During treatment NIR imaging showed zonal progression and SD-OCT scan showed consolidation of the hyperreflective material

A 33 year-old white female presented with central scotoma and distortion in the RE for 2 months. She was 3 months post-partum and nursing at the time of presentation. BCVA was 20/30 in the RE and 20/20 in the LE. Dilated fundus examination showed multiple, well-demarcated zonal areas of outer retinal atrophy at the posterior pole and at the mid periphery of both eyes (Fig. 7). There were pigmented brownish dots located mainly at the margins of the atrophic areas but also within the lesions bilaterally. FAF in both eyes revealed a trizonal pattern and a demarcating hyper-FAF line between the involved and uninvolved retina (Fig. 7); these findings were suggestive of AZOOR [4]. After 2 months, a progression of the zonal lesions was found in both eyes; in the RE, the progression occurred around the disc and towards the fovea and development of a subfoveal type 2 CNV was noted (Fig. 8). Over a course of 3 years the patient underwent intravitreal anti-VEGF injections on a treat-and-extend regimen (12 ranibizumab and 5 aflibercept injections) and her vision has stabilized to 20/40 in the RE. During treatment there was zonal progression at the right macula (Fig. 8). In the LE the zonal lesion at the posterior pole remained stable with fovea sparing and patient remained asymptomatic with a BCVA of 20/20.

### Case 4

**Fig. 9:**
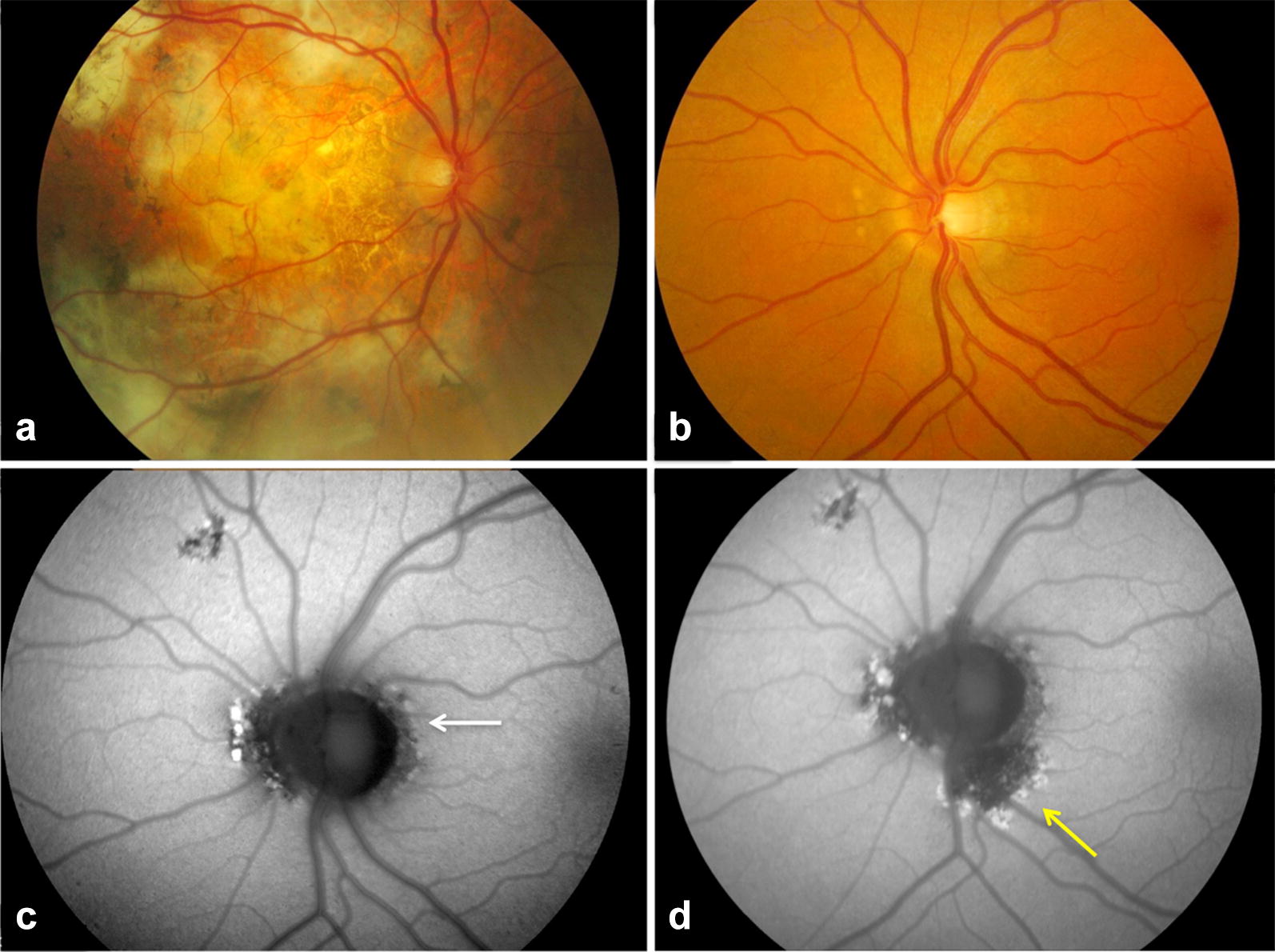
Case 4. **a**, **b** Color fundus photograph (CFP) of AZOOR 10 years after photodynamic therapy in the right eye. **a** CFP of the right eye showed extensive macular scarring. **b** CFP of the left eye showed peripapillary depigmentation and drusen like material. **c** Fundus autofluorescence (FAF) in correspondence of these areas showed a mixed hypo-FAF and granular hyper-FAF signal with a leading hyper-FAF border at the junction between the involved and uninvolved retina (white arrow). **d** After one year of follow-up FAF of the left eye revealed minimal progression of the peripapillary zonal lesion in the left eye (yellow arrow)

In 2004 a 47 year-old white male presented with 2 months history of blurred vision in the RE, associated with photopsias. BCVA was 20/50 in the RE and 20/20 in the LE. Fundus examination of the RE showed a peripapillary lesion. Fundus examination of the LE was unremarkable. FAF of the right peripapillary lesion showed a trizonal pattern and a hyperautofluorescent border between the involved and uninvolved retina [5]. Upon consultation of the recent literature at the time [7], the diagnosis of AZOOR was made. Small haemorrhages were observed at the temporal side of the lesion and fluorescein angiography showed the presence of a choroidal neovascularization. Decision was made to perform a standard PDT with a single spot of 2.5 mm focused on the temporal part of the zonal lesion. Three months after treatment no improvement of visual acuity was recorded. The PDT was not repeated and during follow-up the CNV enlarged resulting in macular scarring. In 2005 there was a central fibrotic pattern with persistent peripheral haemorrhages and subretinal fluid. In the RE BCVA improved from 20/100 to 20/40, with an eccentric viewing; this eye had no longer a reading ability. From 2005 to 2015, a progressive enlargement of the scar was noted in the RE (Fig. 9). In 2009 small peripapillary lesions were found in the LE. FAF features of these lesions were consistent with AZOOR [4]. These zonal lesions increased in size during follow-up (Fig. 9) and remained stable thereafter. At the last follow-up visit in 2015 the left eye eye maintained a BCVA of 20/20, without any symptoms.

## Discussion

AZOOR is a rare condition that occurs most frequently in young females. Presentation may be unilateral or bilateral, with simultaneous or sequential involvement. Prognosis is generally favorable as the central vision is often spared. However, photoreceptor and RPE atrophy make visual field loss recovery quite infrequent [8, 9].

Although the primary lesion seems to be related to photoreceptor outer segment dysfunction [10, 11], its underlying etiology is unknown, and its pathogenic mechanisms remain uncertain. Inflammatory disease has been hypothesized [12], and a history of autoimmune inflammatory systemic disease has been noted in at least 18% of patients [13]. However the benefit of oral steroid treatment for this condition has not been well established. Moreover, some authors have found evidence of disseminated fungal infection [14], and a possible viral etiology has been supported by functional improvement in patients with AZOOR-like illness that were treated with oral valacyclovir [15].

The clinical onset of AZOOR is usually marked by photopsias and acute scotomas, with minimal or no fundus and angiographic changes. The average age at initial presentation is 36.7 years (with a range age of 13 to 79 years), with a predominance in women (76% of cases), and normal fundus appearance in 76% of cases [13]. Our AZOOR cases are therefore atypical with regards to their age at onset, as well as their compounded clinical presentation and course. However, our cases meet the multimodal imaging diagnostic criteria of AZOOR [4] and present with typical autofluorescence and clinical features that are indisputably part of the AZOOR spectrum. The cases presented in this study may be classified as AAOR, described by Gass and Stern as a variant of AZOOR and characterized by a progressively irregular annular band of grey-white deep retinal opacification in a peripapillary location [2, 16]. The most noteworthy aspect of our cases is the concomitance of type 2 choroidal neovascularizations. In our series the mean age of patients was younger than the mean age reported in the literature on AZOOR [13]. It is possible that the mean age deviation of our cases from the mean age reported in the current literature may have contributed to the development of the CNV. Of note our youngest patient (case 3) received more anti-VEGF injections than the other cases. Indeed she was highly discerning for minimal changes of her vision which usually correlated with minimal structural changes on OCT.

CNV is an extremely rare complication of AZOOR [4–6]. To the best of our knowledge only 2 cases of AZOOR complicated by CNV have been described in the current literature [5, 6]. The first case was reported by Cohen and Jampol in 2007 and PDT was used for treating the CNV [5]. Six months after treatment, CNV enlargement and scarring with no progression of the zonal lesion was reported [5]. In the present study we report the long-term follow-up of the case originally described by Cohen and Jampol (case 4). In this case we report a progressive enlargement of the central scar in the treated eye and subsequent mild involvement of the fellow eye with no CNV complication.

Levison et al. [6] recently reported another case of CNV in AZOOR in a 74 year-old female. This patient underwent treatment for bilateral AZOOR with systemic steroids, methotrexate and intravitreal dexamethasone implants. She subsequently developed a CNV in the right eye which was successfully treated with three intravitreal bevacizumab injections. However long-term follow-up was not reported [6].

In the present study we report the multimodal retinal imaging and the long-term clinical course of four patients presenting with AZOOR complicated by choroidal neovascularization, treated with intravitreal anti-VEGF agents (in 3 cases) and with PDT (in one case).

Although VEGF plays an important role in the maintenance of both retinal and choroidal circulations and is one of the key neurotrophic factors, intravitreal anti-VEGF injections presently represent the established treatment for active CNV. In our patients a remission of the choroidal neovascular activity was achieved following administration of anti-VEGF agents. However, zonal or multizonal progression was observed during treatment. Given the short period between the anti-angiogenic treatment and AZOOR progression, a causal relationship between the treatment and the zonal progression could be considered. Indeed, some authors have questioned the safety of VEGF neutralization, and advised cautious administration of intravitreal anti-VEGF. In preclinical studies, after systemic neutralization of VEGF, a significant increase in retinal cell apoptosis in the inner and outer nuclear layers occurred in mice [17]. Moreover, bevacizumab has been reported to neutralize the protective effect of VEGF on retinal ganglion cells from induced oxidative stress [18]. Other authors have raised the issue of ultrastructural toxicity, consisting of mitochondrial disruption in the inner segments of photoreceptors, after intravitreal bevacizumab injections in rabbit eyes [19]. By contrast, multifocal electroretinography has shown no short-term cone photoreceptor toxicity after intravitreal bevacizumab administration [20].

In our cases, it is unclear whether the observed zonal progression was accelerated from anti-VEGF treatment, or is attributable to the natural course of the disease. In case 2, striking progression of the zonal lesions was observed in both eyes during anti-VEGF treatment administration in one eye. This finding might be related to the minimal systemic diffusion of the intravitreal drug and to its possible effect on the fellow eye, as previously hypothesized [21, 22].

The presence of CNV expands the clinical spectrum of AZOOR. While the progressive nature of AZOOR has been well documented [23–26], little is known about the clinical course of AZOOR complicated by CNV.

It is possible that in our series the zonal progression was merely related to the progressive nature of AZOOR in which the CNV was the expression of the underlying active disease.

In our experience, intravitreal anti-VEGF treatment was effective for the stabilization of the CNV complicating AZOOR. However, a concomitant progression of the zonal lesions was observed during treatment. Further studies are required to define the pathogenetic mechanisms underlying this rare association and the role of treatment in the progression of the zonal lesions.
